# Development and Research of Anti-Corrosion Polymer Coatings with Microdefect Blocking Effect

**DOI:** 10.3390/polym18111292

**Published:** 2026-05-25

**Authors:** Svetlana Tyurina, Victor Demin, Vyacheslav Shchelkov, Alexander Ilyin, Sofia Sidorova, Nikita Rashutin, Peter Rusinov

**Affiliations:** 1 Department of Materials Engineering, Advanced Microwave Electronics Engineering School, MIREA–Russian Technological University, 78 Vernadsky Ave., Moscow 119454, Russia; tyurina_s@mirea.ru (S.T.); isingse@gmail.com (S.S.); rashutin@mirea.ru (N.R.); 2 Frumkin Institute of Physical Chemistry and Electrochemistry Russian Academy of Sciences, 31-4, Leninsky Prospect, Moscow 119071, Russia; barsoid@inbox.ru (V.D.); tehnolog@rocor.ru (V.S.); ilinab@list.ru (A.I.)

**Keywords:** corrosion, coatings, polymers, defect self-blocking effect, modification, microencapsulation

## Abstract

Corrosion of materials is a global issue affecting various industries. It leads to a gradual decline in the durability and reliability of materials, resulting in significant economic losses and posing serious risks to human health. To address the challenge of enhancing reliability and durability when materials are exposed to aggressive environments, this study developed new polymer protective coatings. These coatings involve reinforcing an epoxy resin-based polymer matrix with zinc and microencapsulated corrosion inhibitors (activated Al_2_O_3_ + HEDP; activated Al_2_O_3_ + PPA; activated Al_2_O_3_ + ATMP). These microscopic containers encapsulate the corrosion inhibitors. The microstructure of the microcapsules was examined using scanning electron microscopy (SEM) and optical microscopy. Accelerated corrosion tests were performed on the reinforced modified coatings. Coatings reinforced with activated Al_2_O_3_ + HEDP microcapsules demonstrated excellent corrosion resistance in a 3% NaCl solution. In contrast, samples coated with unmodified zinc-filled coatings and coatings modified with Al_2_O_3_ + PPA exhibited the lowest resistance in a 3% NaCl solution. The study also investigated the microdefect-blocking effect in reinforced coatings, which is achieved by filling the pores of the polymer coating with products formed from inhibitor–metal interactions.

## 1. Introduction

**Figure 1:**
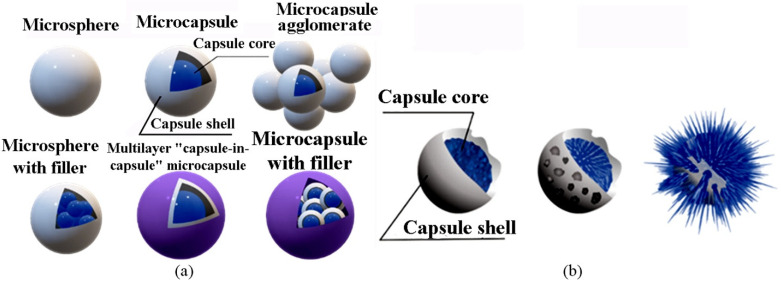
Types of microcapsules—(**a**); functional additive release scheme—(**b**).

Among various corrosion protection strategies, applying polymer coatings to metal surfaces is the most effective and cost-efficient method [1,2]. However, anticorrosive coatings experience changes in their mechanical, chemical, and physical properties over their service life, leading to the formation of microcracks. These microcracks propagate, exposing the metal to atmospheric moisture and oxygen, which accelerates coating peeling and results in defects at the metal/coating interface. Since polymer anticorrosive coatings are composites, the concept of self-healing cracks can be employed to enhance durability [3,4]. Self-healing coatings containing microcapsules can controllably release a healing agent in response to various triggers [5,6,7,8,9,10]. Microencapsulation is the process of encapsulating a functional substance within a protective shell material [11,12,13]; in other words, the capsule is a shell that contains the active substance (Figure 1a,b).

The shell material can be any substance with film-forming properties. The most commonly used materials are organic polymers such as polysiloxanes, polyvinyl alcohol, epoxy resins, polyvinyl chloride, polyvinyl acetate, cellulose derivatives, polycarbonates, and polyurethanes [14,15,16,17,18,19].

The microcapsule allows for the encapsulation of reagents of any type and in any form—liquid, solid, or gas—within its shell. The poorly soluble polymer shell of the capsule ensures a prolonged and uniform release of the functional additives [20,21,22]. Microcapsules can be either open or closed. A closed microcapsule consists of a liquid or solid core enclosed within a polymer shell. An open microcapsule is a solid particle without a shell, characterized by developed porosity and impregnated with an active additive within its pores.

The most common strategy for self-healing coatings involves embedding microcapsules within the coating matrix [23,24,25]. These microcapsules are uniformly distributed throughout the matrix and contain the healing agent in a “trapped” state to prevent any unwanted interaction between the active component and the coating. As illustrated in Figure 1b, when the local environment changes or the active surface experiences mechanical stress, the microcapsules respond by releasing the healing agent to repair the crack.

In recent years, significant attention has been focused on the use of inorganic nanocontainers as carriers of corrosion inhibitors in self-healing coatings. These inorganic nanocontainers primarily include mesoporous nanoparticles such as SiO_2_, TiO_2_, and CeO_2_ and inorganic clays like halloysite, hydrotalcite, and zeolite. Their functionality typically depends on cavities within the nanoparticles, which are used to load both inorganic corrosion inhibitors (such as cerium salts, molybdates, tungstates, vanadates, etc.) and organic corrosion inhibitors (such as imidazoline (IMI) and benzotriazole (BTA), etc.). The corrosion inhibitor, released from the micro- or nano-container in response to environmental exposure, physically or chemically interacts with the alloy to prevent further electrochemical corrosion, thereby enabling the self-healing of the coating. Zinc-containing polymer coatings have proven highly effective in protecting metals from corrosion through electrochemical (cathodic) and sacrificial protection. However, several inherent disadvantages of this type of anti-corrosion coating should be noted:

- Zinc-containing polymer coatings block the penetration of anions but are permeable to cations, which leads to delamination of the coating;
- Service life is limited by the time required for dispersed metal particles to dissolve.

The incorporation of an inhibitor into a polymer coating, either as a functional additive or within microcapsules, can solve a number of technological challenges. These include extending the coating’s service life, enhancing corrosion resistance without reducing the heat transfer coefficient, and enabling the addition of other performance properties, such as antiscale effects [26]. In this case, both microcapsule structure and materials for its components (the shell, the inert carrier, and the functional agent, which in our case are microencapsulated corrosion inhibitors (MCI)) play a crucial role. The combinations and relative arrangements of these structural elements are diverse; the literature reports successful use of multi-walled [27], multi-core [28], open or shell-less [29], and carrier-free soft microcapsules [30]. Studies [31,32] have demonstrated that phosphonic acids are promising candidates for incorporation into polymer matrices, as they can be effectively desorbed through the microcapsule wall and subsequently reach the coating surface. Microencapsulation technology isolates MCI from polymer resins and hardeners during the curing stage, enabling the formation of a uniform coating base and preventing negative effects on coating adhesion [33]. A critical challenge remains the selection of an inert carrier that ensures sufficient loading of MCI into the microcapsule and its effective release when the coating is exposed to aggressive environments. In this context, methods for optimizing the properties of zinc-containing polymer coatings are being developed. In this study, we investigated the potential for modifying zinc-containing, highly filled (89% by weight) polymer coatings using microcapsules loaded with corrosion inhibitors.

The aim of this study is to develop novel polymer composite protective coatings reinforced with microencapsulated corrosion inhibitors that possess microdefect-blocking properties, thereby enhancing the durability and reliability of materials exposed to aggressive environments.

## 2. Materials and Research Methods

Hydroxyethylidene diphosphonic acid (HEDP) (Ruskhim, Moscow, Russia), phenylphosphonic acid (PPA) (Acros Organics, Geel, Belgium), and nitrilotrimethylphosphonic acid (ATMP) (Akvakhim, Moscow, Russia) were used as MCI. The characteristics of the corrosion inhibitors used as modifying additives are presented in Table 1.

Activated Al_2_O_3_ (Khromlab, Moscow, Russia) was used as an inert carrier. It is a highly active sorbent with a porous structure, commonly used for drying media and as a catalyst support. The powder particle size is 0.5–20 µm. S_surf_ > 200 m^2^/g.

ED-20 epoxy resin (Ruskhim, Moscow, Russia) was used as the shell material. Industrial amine-curing composites were also employed as shells and polymer coating matrices, including: 017—zinc-protective primer; 5095—waterborne epoxy; 793—solvent-borne epoxy–phenol-formaldehyde–furan; and ED—undiluted model epoxy (SPA “ROKOR” Russia).

The design of the functional self-healing core is based on the strong chelating ability of phosphonic acids toward iron and zinc ions, which drives the localized formation of stable protective complexes. Activated Al_2_O_3_ was selected as the core carrier due to its developed porous structure and high specific surface area, ensuring maximum capillary loading and chemical compatibility with the inhibitor payload. The microcapsules were synthesized via a multi-step interfacial polymerization process to form a protective epoxy shell. To preserve commercial and intellectual property rights, the detailed chemical synthesis parameters and technological conditions follow the patented method [34]. The volume fraction of the microcapsules embedded into the baseline zinc-rich epoxy matrix was optimized at 1% wt.%, serving as the design threshold to guarantee an effective self-healing response without compromising the baseline mechanical and barrier cross-linking density of the polymer film.

Microcapsules based on the above-mentioned carriers and inhibitors were synthesized using dry milling and thermostatting to impregnate the anti-corrosion agent into the pores of the inert carrier. The resulting inhibitor-loaded particles were coated with a polymer shell through ultrasonic dispersion in epoxy resin, followed by heat curing. The microcapsules were then cleaned of excess resin and dried. To estimate the amount of functional filler released from the microcapsules and to plot kinetic curves, ref. [35] (corresponding to ISO 6878:2004) was used. Its practical application is comprehensively discussed in [36]. An SF PE-5400 UV spectrophotometer (EKROSKHIM LLC, Saint Petersburg, Russia) equipped with a red filter (λ = 670 nm) and 10 mm quartz cuvettes was used. Phosphate ions were quantitatively determined using a colorimetric method, which produces a blue phosphomolybdenum complex formed by the reaction of phosphate ions with ammonium molybdate in an acidic medium in the presence of a reducing agent. A solution of tin (II) chloride in glycerol was used as the reducing agent. The oxidation of phosphonates to phosphates was performed by boiling the solution with ammonium persulfate in an acidic medium. To evaluate particle morphology, an Altami POLAR 3 polarizing microscope (Altami LLC, Russia) was used for transmitted and reflected light studies employing bright field and polarization techniques.

Scanning electron microscopy and X-ray microanalysis of microcapsule and polymer coating samples were performed with an SM-32A scanning electron microscope (Melytec, China) with an Xplore 30 X-ray detector.

The resulting microcapsules were incorporated into an industrial epoxy zinc-filled primer coating 017. In all cases, the microcapsule content was 1%. The comparison sample did not contain microcapsules. This primer coating was used for accelerated testing of composite coatings for resistance to climatic factors and for electrochemical impedance spectroscopy (EIS).

Accelerated testing for resistance to climatic factors was conducted in accordance with GOST 9.401-2018 (corresponding to ISO 4628-8:2012), to the GOST 9.401-2018 Steel 3(calm) plates were cleaned and degreased using acetone. The coating was applied with a brush to all sides of the plate. Coating thickness was measured using an MT-2007M thickness gauge (LLC ITC “AKA-SCAN+”, Moscow, Russia). The coating thickness was 70 ± 5 µm. An X-shaped notch was made on the samples. The plates were exposed to 3% NaCl for 91 days.

The AZtecOne, version 5.0 (Abingdon, England) software was used to accumulate and process the X-ray spectra. All photographs and measurements were performed at an accelerating voltage of 15 kV. The beam current on the sample was about 2 nA in the microanalysis mode and 30–40 nA when obtaining raster images. The study of the samples by X-ray phase analysis was performed on a Tongda TD-3700 X-ray diffractometer (Dandong Tongda Science & Technology Co Ltd., China). The structure of the samples was studied by the AWACS method (θ–2θ scheme) in CuKα radiation (wavelength λ = 0.0154 A, beam size on the sample 0.6 × 10 mm) with scanning by incidence angle θ in the range of angles 2θ = 4–140° with a step of 0.0191° and a counting time of 0.2 s at each step. The total scanning time was approximately 15 min.

To determine the electrochemical properties of polymer protective coatings, tests were conducted in accordance with the ISO 16773 “Paints and varnishes—Electrochemical impedance spectroscopy (EIS) on high-resistivity coated specimens.” An IPC-Pro Mf potentiostat–galvanostat (OmLiber-Science, Russia) equipped with an FRA electrochemical impedance measurement module was used for measurements. The three-electrode circuit included a silver chloride reference electrode, a platinized auxiliary electrode, and a working electrode (test specimen). The test specimens were St3sp metal plates with a 50 mm × 50 mm × 2 mm coating. All plates were cleaned and degreased with acetone before application. The coating was applied with a brush on one side of the plate. The coating thickness was 70 ± 5 μm. Each sample was measured at least three times to ensure reproducibility. Impedance measurements for the polymer-coated samples were performed at frequencies ranging from 0.1 to 90,000 Hz. Exposure was conducted in a 3% NaCl environment for 91 days.

**Figure 2:**
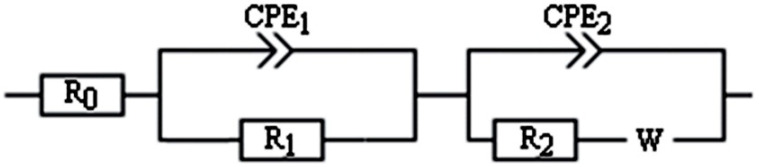
Voight’s Circuit.

The resulting EIS spectra were analyzed using DCS (Dummy Circuit Solver) software to select the optimal equivalent circuit that best represents all samples throughout the entire exposure period. For this purpose, calculations were performed using the Voigt circuit diagram shown in Figure 2. The CPE (constant phase element) models the capacitance of the electrical double layer, R represents the charge transfer resistance, and W denotes the Warburg impedance associated with diffusion processes. The first, part of the circuit characterizes the behavior of the polymer coating and corresponds to high-frequency processes. The second, or “internal” part of the system describes low-frequency Faradaic processes, which, in this case, correspond to corrosion at the metal surface interface [37].

This circuit includes a constant phase element (CPE) to account for the non-ideal nature of the capacitive elements. The impedance of this element includes the phase factor n; for n = 1, the CPE acts as an ideal capacitor, for n = 0.5, as a Warburg diffusion element, and for n < 0.5, it exhibits primarily resistive properties. Thus, the value n of the constant phase element indicates the degree of the system homogeneity.

The amount of released inhibitor was determined by preparing colored samples containing HEDP + Al_2_O_3_ (activated) microcapsules and measuring the optical density of these solutions over 1008 h. The full methodology is described in [38].

## 3. Results and Discussion

### 3.1. Structural Analysis

**Figure 3:**
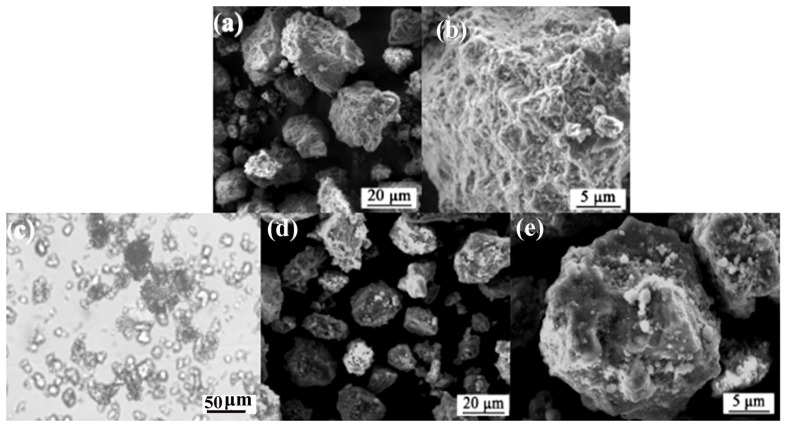
Microcapsules of the HEDP + Al_2_O_3_ composition in a glycerol oil medium—(**a**); appearance of microcapsules obtained on activated aluminum oxide at a resolution of 20 μm (**b**) and 5 μm (**c**); SEM images of the microcapsules—(**d**,**e**).

According to optical microscopy, the resulting microcapsules (5–50 µm) generally appear as translucent, irregularly shaped particles with uneven surfaces and attached satellite particles. Due to their morphology, they are prone to agglomeration but readily disintegrate into individual particles in a liquid medium (Figure 3c).

Figure 3d,e show SEM images of the microcapsules. The capsules have smoother surfaces, free of edges and chips, compared to the surface of the particles of the original inert carrier (Figure 3b). The polymer shell contains small satellite particles that are clearly and securely attached (Figure 3e). The smaller size of the microcapsules is due to the preliminary milling of Al_2_O_3_ prior to synthesis.

**Figure 4:**
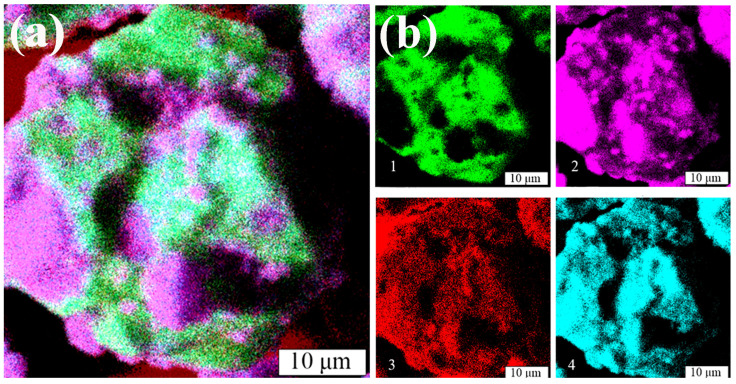
EDS map of the microcapsule surface: (**a**)—multilayer image, (**b**)—elemental image (1—phosphorus, 2—aluminum, 3—carbon, 4—oxygen).

Elemental mapping (Figure 4) reveals that the characteristic HEDP inhibitor, phosphorus, is distributed across the microcapsule surface, with a higher concentration in the recesses. The presence of phosphorus in the polymer shell indicates that HEDP participates in the curing process of the epoxy resin. Oxygen and aluminum are components of the inert carrier.

**Figure 5:**
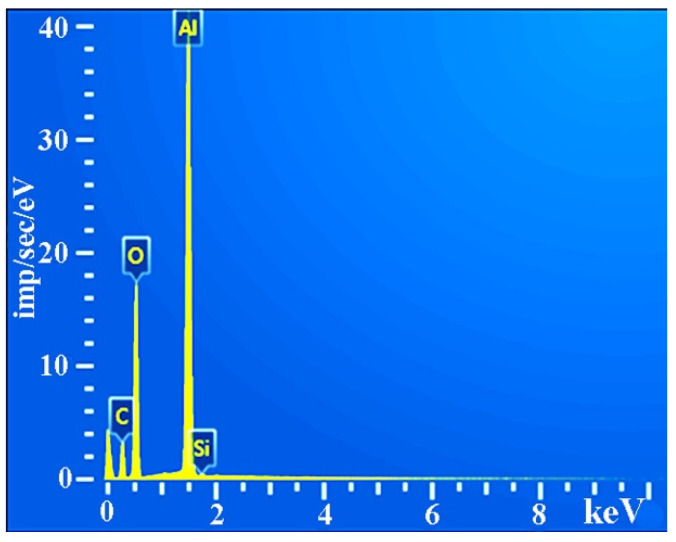
Energy-dispersive spectrum of HEDP+ Al_2_O_3_ microcapsules (activated).

The elemental composition of the microcapsule surface and the corresponding spectrum are shown in Figure 5 and Table 2.

Aluminum and oxygen are the primary components of the inert carrier material. The presence of carbon acts as an indicator of the adhesion between the epoxy shell material and the capsule. A small amount of silicon is present due to contamination (trace amounts). After identifying the most suitable carrier for microcapsule production, we conducted accelerated corrosion tests on zinc-filled primers modified with Al_2_O_3_-based microcapsules containing various corrosion inhibitors.

### 3.2. Corrosion Testing

**Figure 6:**
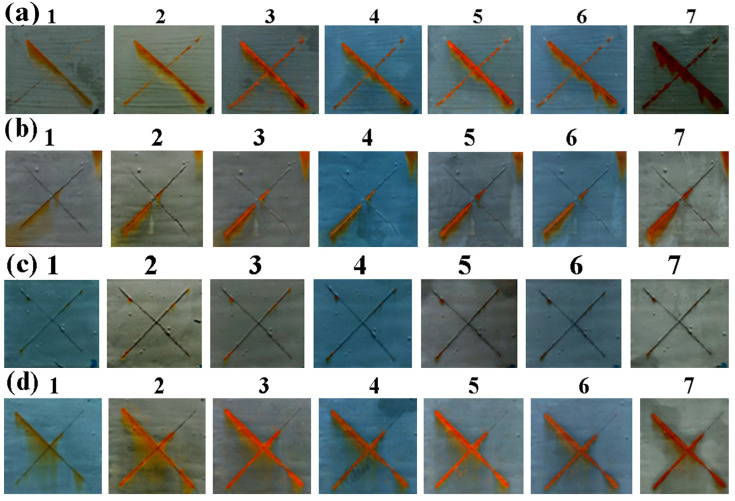
Results of accelerated corrosion tests (time of testing: 1—0 h, 2—24 h, 3—168 h, 4—192 h, 5—336 h, 6—458 h, 7—2162 h): (**a**) unmodified material; (**b**) material modified with Al_2_O_3_-based microcapsules impregnated with a corrosion inhibitor (ATMP); (**c**) material modified with Al_2_O_3_-based microcapsules impregnated with a corrosion inhibitor (HEDP); (**d**) material modified with Al_2_O_3_-based microcapsules impregnated with a corrosion inhibitor (PPA).

The test results are presented in Figure 6. The coatings modified with microcapsules exhibited lower levels of iron oxide formation compared to the reference sample. Additionally, the reference sample showed a gradual increase in iron oxide formation over time, whereas the coatings containing microcapsules maintained nearly constant levels after 14 days of exposure.

Figure 6 shows that samples modified with Al_2_O_3_ + HEDP microcapsules (Figure 6c) exhibit good corrosion resistance in a 3% NaCl solution. Meanwhile, samples coated with an unmodified zinc-filled coating (Figure 6a) and a coating modified with PPA (Figure 6d) demonstrated the lowest corrosion resistance. These samples exhibited active corrosion processes on their surfaces.

**Figure 7:**
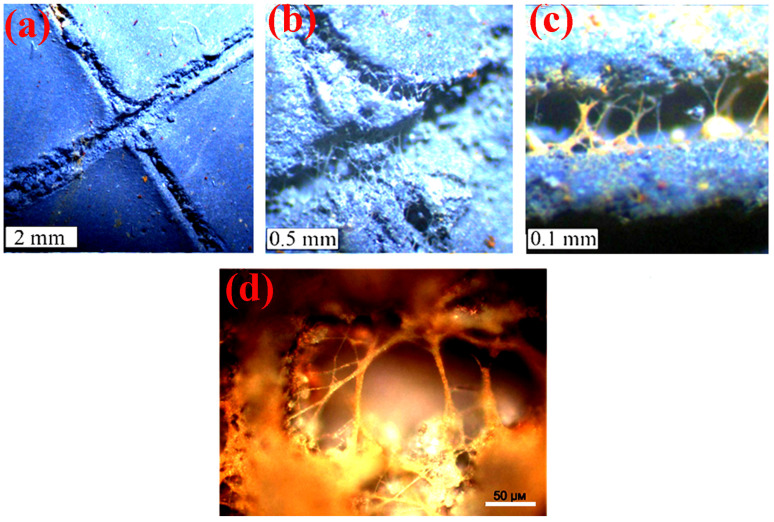
Filament-like structures on a sample of a coating containing HEDP + Al_2_O_3_ microcapsules (activated)—(**a**–**c**); photographs of cuts on samples painted with a modified zinc primer composition after exposure to a 3% NaCl solution in reflected light using the bright field and polarization method—(**d**).

After 400 h of exposure, the coating modified with HEDP + Al_2_O_3_ microcapsules (activated) showed the appearance of randomly distributed filiform structures at the sites of coating damage (Figure 7a–c). Reflected light studies of the samples using the bright field and polarization methods (Figure 7d) showed that the formed fibers constitute an organopolymer framework.

An assessment of the stability of the formed filamentary structures indicates that the filaments remain stable and continue to grow during continuous exposure to a 3% NaCl solution at +50 °C for 100–1000 h. No structural degradation is observed in the salt solution during daily temperature cycling between +20 °C and +50 °C. Additionally, no filament dissolution occurs under the operating conditions of ion-exchange filters (+30 °C) with technologically controlled water/salt solution changes. The durability of the filaments is preserved when large-scale equipment is dried for 24–96 h during preventive inspections, and the coating is exposed to air at temperatures ranging from +5 °C to +35 °C.

**Figure 8:**
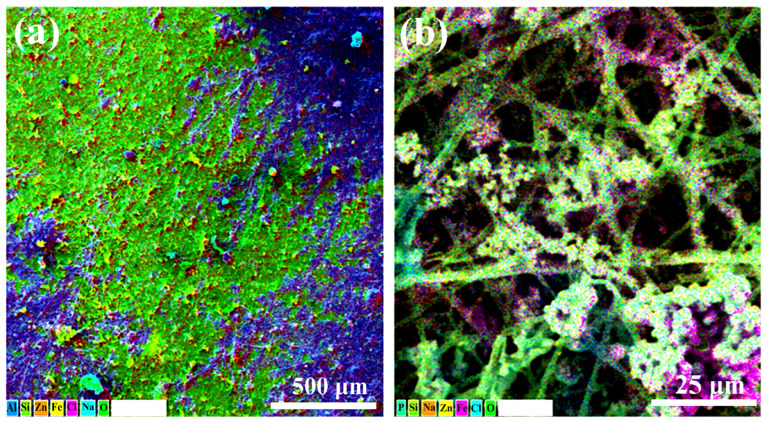
Multilayer EDS map of the plate surface after accelerated corrosion testing—(**a**,**b**).

Mapping the distribution of the characteristic elements of the inhibitor, specifically phosphorus P, in the sample was visualized using energy-dispersive X-ray spectroscopy (EDS). EDS mapping of the coating material after testing (Figure 8a,b) showed that the threads are distributed across the entire surface, not just at the damaged sites. Phosphorus is present on the surface of the threads, indicating the formation of insoluble products resulting from the interaction of HEDP with the metal in the system. This compound is presumably identified as (1-hydroxyethylidene) iron (II) diphosphonate, which is the most widely described insoluble or poorly soluble HEDP complexes with metals [39,40,41,42].

The microdefect blocking effect is achieved by filling the pores of the polymer coating with inhibitor–metal interaction products. The presence of these products in the system is confirmed using SEM-EDS. They appear on the sample surface as fibers containing phosphorus atoms (Figure 8), a characteristic element of HEDP and its compounds. The presence of poorly soluble corrosion products within the bulk material and their influence on the anticorrosive properties of the coating are demonstrated by analyzing the elements of the equivalent circuit of the process (Figure 2) and by monitoring changes in their values over time (Figure 6). The resistance of the sample modified with microcapsules was orders of magnitude higher than that of the reference sample, indicating a significant increase in protective properties. The CPE factor of the modified coating increased during exposure, suggesting that the system became more homogeneous. However, the capacitance remained consistently low, indicating that the coating containing HEDP + Al_2_O_3_ (activated) does not accumulate water. The Warburg diffusion impedance remained consistently high, indicating the monolithization of the coating and the inhibition of corrosion by diffusion processes that slow its progression. In contrast, the comparison sample showed a decrease in this indicator during exposure due to the formation of looser corrosion products and direct contact of aggressive ions with the metal surface.

### 3.3. Inhibitor Release Kinetics from Microcapsules

**Figure 9:**
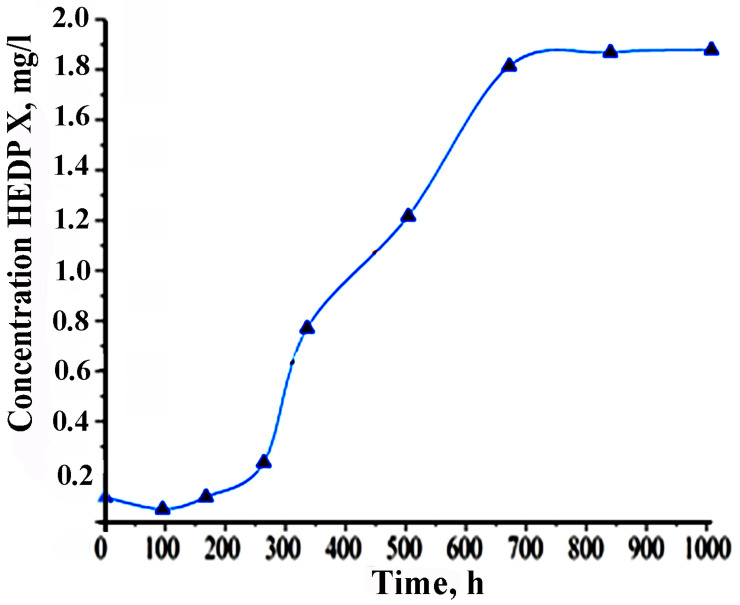
Kinetics of HEDP release from microcapsules.

The graph (Figure 9) shows that capsules based on activated Al_2_O_3_, due to the porous surface of the carrier. Additionally, the release time was twice as long as that of the other aluminum oxides tested. Based on these results, activated Al_2_O_3_ was selected as the most effective carrier, and further studies were conducted on microcapsules using it.

In the next stage of the study, the microcapsules were incorporated into a model epoxy composite to study HEDP diffusion through both the shell and the polymer matrix. When the water front diffusing into the epoxy composite reaches the region containing the phosphonate inhibitor within the microcapsule, a phosphonate solution begins to form inside the MCI. This is followed by its desorption and diffusion from the MCI into the polymer matrix of the coating layer. In the case of shell-based MCI, diffusion initially occurs through the MCI shell.

**Figure 10:**
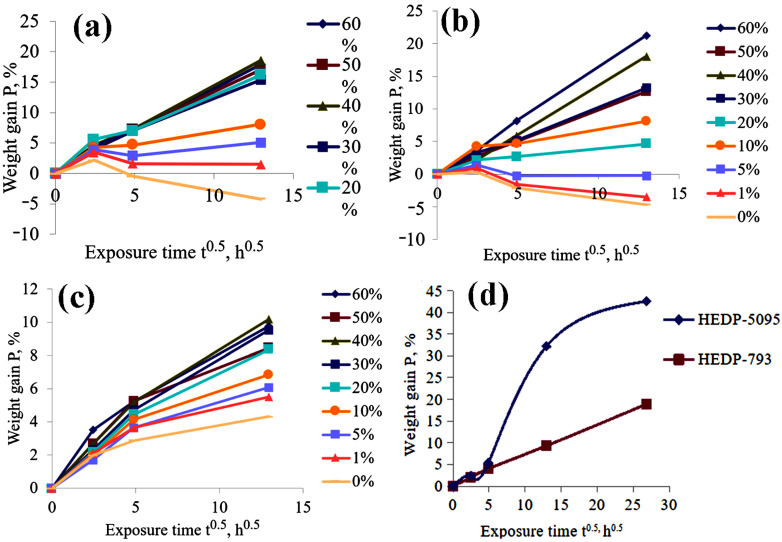
Kinetics of weight gain of composites: (**a**)—5095 in 1–60% HEDP solutions at +96 °C; (**b**)—793 in 1–60% HEDP solutions at +96 °C; (**c**)—ED in 1–60% HEDP solutions at +96 °C; (**d**)—5095 and 793 in 60% HEDP solution at +96 °C.

Both the MCI shell and the polymer matrix of the coating are composed of epoxy composites 5095, 793, and ED (all samples from this series No. 1 were cured at +20 °C for 168 h and then at +96 °C for 6 h). Figure 10a–c illustrate the kinetics of material weight gain at +96 °C in HEDP solutions, ranging from pure water of 0% and up to a 60% concentration, which corresponds to a saturated solution. The choice of the solution temperature of +96 °C was motivated by the potential application of epoxy composites in anti-corrosion coatings, not only for equipment exposed to oil, oil products, bottom sediments, mineral, or seawater but also for hot water at temperatures between 95 °C and 107 °C, including drinking water. As shown in Figure 10a–c, for the model, composite ED exhibits significantly lower sorption of HEDP solutions (up to 10%) compared to the industrial grades 5095 and 793 (18–22%). This suggests that both epoxy composite 5095 and epoxy–phenol-formaldehyde–furan 793 are suitable for facilitating rapid transfer of phosphonates from the MCI to the protected substrate, thereby providing high protective concentrations of the inhibitor on the metal surface.

For an exposure time of 6 h (2.45 h^0.5^), a sudden increase in material weight is observed. This is followed by a transition to linearity in the coordinates P~√t, which is typical for the sorption of acids [43], including phosphonic acids with amine-cured epoxides. For HEDP, this behavior is especially evident at solution concentrations of 30–60% (Figure 10a–c). High-concentration solutions of 60% HEDP are shown in Figure 10d. In this case, unlike series No. 1, samples of composite 5095 (series No. 2) were cured at +20 °C for 168 h without hot curing, while samples of composite 793 were additionally cured at +120 °C for 4 h. Up to a solution diffusion time of 168 h (13 h^0.5^), a sudden release, or “jump”, is observed for composite 5095 during inhibitor leaching from MCI. After this point, the release rate becomes comparable to the sorption rate (slope tangents are close) of composite 793 (Figure 10d). It can be concluded that for HEDP, it is technologically straightforward to vary sorption values (10–40%) or inhibitor release concentrations (1–2 mg/L) on the surface within controlled limits by optimizing the composition of the epoxy shell and/or matrix or their curing modes.

From a practical point of view, it is important that the required MCI optimization parameters can be obtained using industrial cold-curing composites [44] used to protect expensive large-scale oil and petroleum product storage tanks with volumes of 1000–100,000 m^3^.

### 3.4. Electrochemical Impedance Spectroscopy

**Figure 11:**
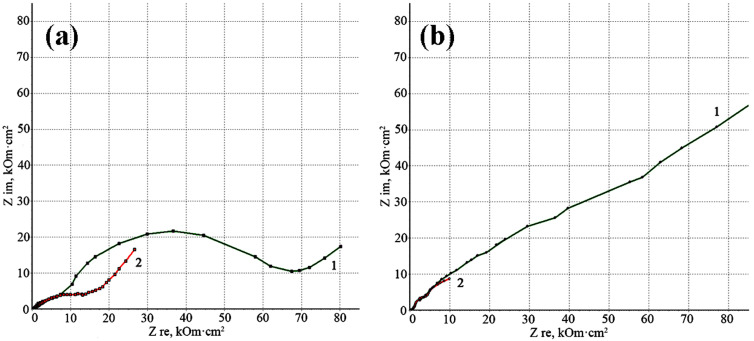
Hodograph for a blank sample and coatings with microcapsules (1—HEDP + Al_2_O_3_ (activated), 2—comparison sample): (**a**)—after 1 day; (**b**)—after 91 days (exposure to 3% NaCl).

A study of the impedance of zinc-protective primer coatings modified with HEDP + Al_2_O_3_ (activated) microcapsules revealed that both initially and after 91 days of exposure (Figure 11), the modified coating demonstrated higher impedance than the reference sample across the entire frequency range. A decrease in the average impedance across the entire frequency range was observed for the reference sample, in contrast to the sample containing HEDP + Al_2_O_3_ (activated) microcapsules. This phenomenon may be related to the gradual impedance of the coating and the diffusion of sodium chloride ions. The similar shape of the hodographs (Figure 11a) suggests that the initially introduced microcapsules do not alter the mechanism of protective action but only affect the intensity of the processes occurring in the coating.

**Figure 12:**
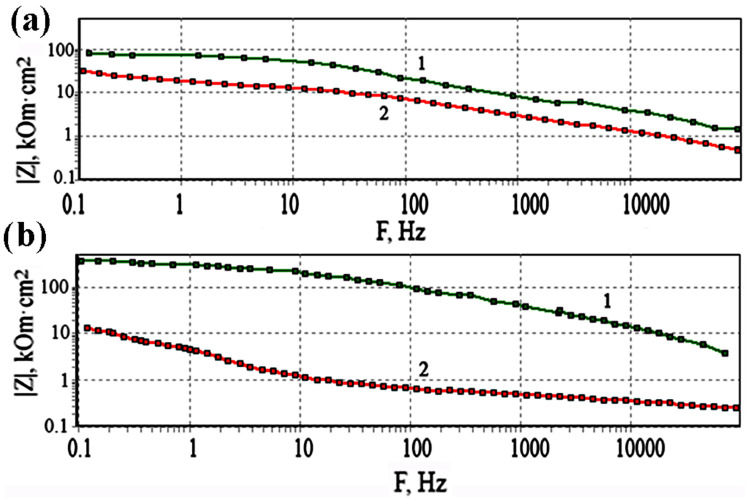
Impedance modulus for the comparison sample and coatings with microcapsules (1—HEDP + Al_2_O_3_ (activated), 2—comparison sample): (**a**)—after 1 day, (**b**)—after 91 days (exposure to 3% NaCl).

Since exposure, the impedance of the coating modified with microcapsules was an order of magnitude higher than that of the comparison sample. Figure 11b shows only a portion of the hodograph at current frequencies up to 1000 Hz, while Figure 12 presents the impedance modulus values over the entire frequency range.

To contextualize the performance of the developed self-healing system, its low-frequency impedance modulus (|Z|_0.01 Hz_) was compared with values reported for smart and modified epoxy coatings. In [45], a zeolitic imidazolate framework-8 (ZIF-8) was employed as a nanocontainer for loading benzotriazole, enabling an increase in the coating’s |Z|_0.01 Hz_ from 10^3^ Ω·cm^2^ to 10^4^ Ω·cm^2^. Building upon this approach, the authors of [46] replaced the inhibitor with 8-hydroxyquinoline (8HQ) and surface-modified the containers with polydopamine, which enhanced the impedance by two orders of magnitude. Furthermore, Su et al. [47] modified highly zinc-filled epoxy coatings with a smart conductive filler composed of polyaniline (PANI) and reduced graphene oxide (rGO) treated with HEDP. After 70 days of immersion, their |Z|_0.01 Hz_ was nearly an order of magnitude higher than that of the HEDP-free control sample. In the present study, the low-frequency impedance modulus increased significantly from approximately 15 kΩ·cm^2^ to over 370 kΩ·cm^2^ upon the integration of the HEDP + Al_2_O_3_ (activated) microcapsules. This performance after prolonged exposure to 3% NaCl electrolyte demonstrates that the developed primer is competitive and aligns perfectly with global achievements in smart protective materials.

**Figure 13:**
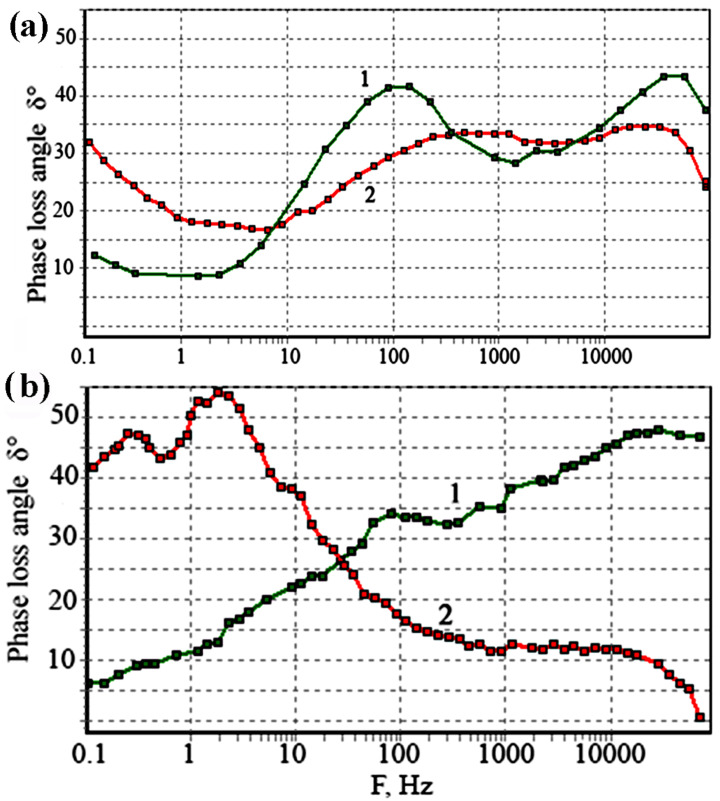
Bode plots for the sample and coatings with microcapsules (1—HEDP + Al_2_O_3_ (activated), 2—comparison sample): (**a**)—after 1 day, (**b**)—after 91 days of exposure to 3% NaCl.

The analysis of the Bode plots (Figure 13) indicates that anodic processes occur in the original primer coating, both initially and after exposure, as evidenced by the increase in the low-frequency curve. Since the composite coating under study is zinc-filled, this process likely corresponds to zinc oxidation. In the microcapsule-modified sample, this process nearly ceased after 91 days of exposure. The high-frequency region reflects the coating’s protective properties. The largest phase loss angle (44°) indicates that the coating containing microcapsules exhibited a high surface capacitance, which was maintained throughout the exposure period. In contrast, the protective properties of the comparison sample degraded, as shown by the decrease in the phase loss angle from 30° to 10°.

The hodographs and Bode plots obtained during the exposure were analyzed using DCS software with the Voigt model (Figure 2). The calculated values of the scheme elements are presented in Table 3. The average calculation error was 2.9%. The data’s correspondence to the sequential scheme serves as an indication of the good protective properties of the coatings under study (in contrast, for example, to the Mansfeld scheme, which describes systems with through defects).

Next, the elements of the Voigt sequential scheme were examined. They were conventionally divided into an “external” block, characterizing the resistance R_1_ and capacitance CPE_1_ of the polymer layer, and an “internal” block, describing the processes at the interface of the metal substrate or zinc particles. The resistance R_1_ of both modified and unmodified coatings with microcapsules increased up to 21 days of exposure, which may be attributed to the clogging of pores by zinc corrosion products. The low value of this parameter is generally characteristic of systems with a high zinc content. A subsequent decrease in this parameter indicates degradation of the polymer matrix. However, throughout the entire exposure period, R_1_ for the coating with HEDP + Al_2_O_3_ (activated) remained higher than that of the reference sample. This indicates that the introduction of microcapsules did not compromise the integrity of the polymer matrix and did not lead to the formation of microdefects or cracks at the polymer–capsule interface. The increase in the CPE_1_ capacitance of the comparison sample during exposure indicates water absorption by the polymer, as the dielectric constant of water (≈80) is much higher than that of epoxy resin (≈3–4). For both systems, a sharp drop in capacitance is observed on the 91st day of exposure (0.075 and 0.007 μF·cm^2^, respectively). This must be considered alongside the “internal” part of the system. In the comparison sample, both the CPE1 capacitance and R_2_ decrease, which often indicates delamination or the formation of large cavities. Conversely, in the sample with HEDP + Al_2_O_3_ (activated), there is an explosive increase in R_2_ to 363.7 kΩ·cm^2^, signaling the blocking of microdefects. Using the n factor of CPE_1_, as mentioned earlier, it is possible to evaluate the homogeneity of the coatings. As shown by the data, the introduction of HEDP + Al_2_O_3_ microcapsules (activated) significantly improves coating uniformity by blocking microdefects and filling pores with corrosion products, which increase during exposure. For the comparison sample, the n CPE_1_ decreased from 1 to 0.45, indicating a transition to through-diffusion. In contrast, for the coatings with microcapsules, this value increased from 0.57 to 1, effectively turning the coating into a capacitor [47]. The decrease in n CPE1 of the comparison sample corresponds to an increase in coating heterogeneity that becomes evident during exposure. The increase in CPE1 n to 1.00 on the 91st day of exposure is attributed to coating delamination from the substrate. Iron oxides and blisters in the polymer coating were also visually observed on the sample. Similar defects were noted during accelerated corrosion testing (Figure 6a). The electrolyte penetrating the large cavities thus created a capacitor effect corresponding to this CPE value [48]. When analyzing the “internal” part of the system, the differences between the coatings become apparent. For the comparison sample, R_2_ decreases from 14.3 to 7.1 kOm·cm^2^, while capacitance and the CPE_2_ factor increase, indicating charge accumulation on the metal surface. For the sample with microcapsules, a significant increase in R_2_ is observed, accompanied by consistently low capacitance and a CPE_2_ factor value close to 0.5, suggesting the influence of diffusion processes on the corrosion reaction rate at the metal surface. The resistive barrier effect, observed as an increase in R_2_, is accompanied by active diffusion-controlled processes, specifically the release of the inhibitor from the microcapsules. This is reflected in the observed CPE_2_n value, which decreases from 0.68 to 0.49, approaching the ideal behavior predicted by the Warburg model. Analysis of the Warburg element W also allows us to evaluate the significant contribution of diffusion processes to the corrosion protection of the systems under study. The importance of this parameter decreases in the case of an unmodified coating, as the formation of more friable corrosion products during exposure increases the coating’s permeability, enabling ions from the aggressive environment to come into direct contact with the metal [49]. Coatings containing HEDP + Al_2_O_3_ (activated) microcapsules consistently exhibited a high value of this parameter, approximately 17 kOm·cm^2^/s^0.5^ with peaks of 27–32 kOm·cm^2^/s^0.5^ on days 2 and 15. This behavior may indicate the active release of the inhibitor during this period. These findings generally align with the release profile of HEDP from microcapsules (Figure 9), where active diffusion of the inhibitor through the polymer shell began on the twelfth day.

To evaluate the overall corrosion barrier performance of the developed coatings, the total polarization resistance R_p_ was calculated as the sum of the individual resistance elements R_1_ and R_2_. The value of R_p_ serves as a primary indicator of the overall kinetic resistance to electrochemical and corrosion processes at the interface, in accordance with the design principles discussed by Yang et al. [50]. For the unmodified reference coating, R_p_ dropped significantly by the end of 91 days of exposure, from 14.6 to 6 kOhm·cm^2^, indicating severe degradation and electrolyte penetration. In contrast, the coating containing microcapsules demonstrated a sharp increase in R_p_, reaching 371 kOhm·cm^2^. This massive rise in polarization resistance explicitly confirms that the released HEDP inhibitor successfully passivated the substrate, providing long-term active protection efficiency.

**Figure 14:**
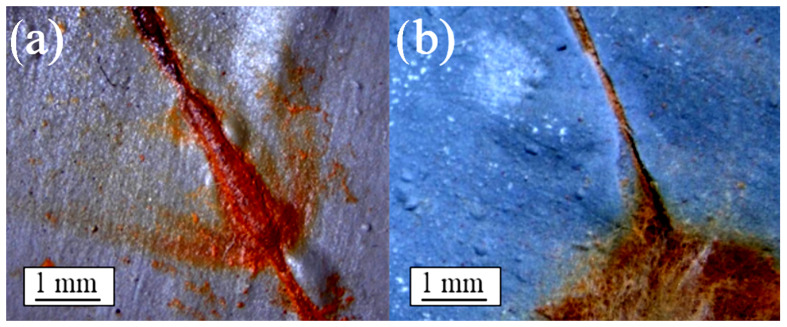
Surface morphology of the coating after accelerated corrosion tests: (**a**)—comparison sample, (**b**)—coating with HEDP + Al_2_O_3_ (activated) microcapsules.

Optical microscopy observations of the coating surfaces after 91 days of exposure (Figure 14) fully correlate with the EIS data.

The reference sample (Figure 14a) exhibits severe matrix degradation, characterized by deep cracks, osmotic blistering, and heavy accumulation of loose iron oxide. This morphologically supports the drastic drop in and the transformation of the coating into a porous, heterogeneous pathway for through-diffusion. In contrast, the microcapsule-containing coating (Figure 14b) demonstrates high integrity. The damaged sites are densely populated with stable, interconnected filiform organopolymer structures (iron phosphonates). These optical findings provide direct visual evidence of the self-blocking effect, explaining the monolithization of the matrix, the absence of water accumulation, and the restoration of ideal capacitor-like behavior.

## 4. Conclusions

As a result of this study, a technology was developed for synthesizing closed-loop microcapsules based on HEDP + Al_2_O_3_ (activated). Zinc-filled epoxy primer coatings modified with these functional particles were produced, and their anticorrosive properties on steel substrates were evaluated. It was demonstrated that, under aggressive environmental conditions, the modified coatings release MCI, which alters the composition and structure of the resulting iron oxides. Incorporating microcapsules into the primer coating reduces the amount of oxides and coating defects. The introduction of functional particles into the polymer matrix increased the phase loss angle in the high-frequency region after exposure from 10° to 47°, indicating enhanced protective properties of the coating. Additionally, the inclusion of microcapsules improved the impedance of primer coatings across the entire frequency range. Specifically, HEDP and activated Al_2_O_3_ particles increased the impedance Z from 0.1 to 380 kOm in the low-frequency range, effectively inhibiting corrosion processes on the steel substrate. For HEDP-based MCI, it is technologically straightforward to vary sorption values (10–40%) or inhibitor release concentrations (1–2 mg/L) by optimizing the composition of the epoxy shell and/or matrix or their curing modes. The desired MCI optimization parameters can be achieved using industrial cold-curing composites commonly employed to protect large-scale oil and petroleum product storage tanks.

The study identified the optimal inert carrier and the most effective corrosion inhibitor for protecting steel surfaces against corrosion. Modifying the zinc-filled composition with Al_2_O_3_-based microcapsules saturated with HEDP, a corrosion inhibitor, significantly reduced the corrosion rate. Additionally, exposing steel samples coated with the modified composition to a 3% NaCl solution led to the formation of organopolymer fibers on their surfaces, which helped block surface defects and enhance durability.

The following are application scenarios for protection using self-healing coatings containing microencapsulated corrosion inhibitors:

- In the oil and gas industry, the protection of equipment, structures, and pipelines from corrosion during oil and gas production, transportation, and processing from exposure to acidic and saline waters and solutions.
- In the thermal and nuclear power industries, the protection of water treatment and heating equipment, including the inner surface of steam condenser heat exchange tubes.
- In cold and hot municipal water supply systems and the food industry, the prevention of internal and external corrosion of pipes, valves, and equipment, including those in contact with drinking water and food and beverage products.
- In the chemical and mineral fertilizer industries, the protection of equipment from organomineral acidic, saline, and oxidizing aggressive environments.

## Figures and Tables

**Table 1:** Characteristics of functional additives for microencapsulation.

№	Name of the Functional Additive	Formula	Characteristics
1	Hydroxyethylidene diphosphonic acid (HEDP)	C_2_H_8_O_7_P_2_ 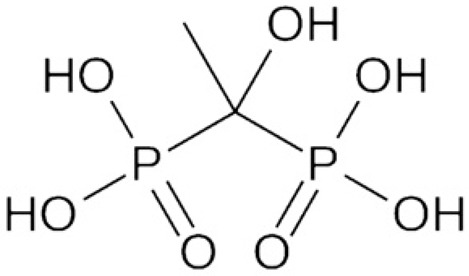	Molecular weight: 206.03 Melting point: 198–200 °C Density: 1.4–1.45 g/mL at 20 °C
2	Phenylphosphonic acid (PPA)	C_6_H_7_O_3_P 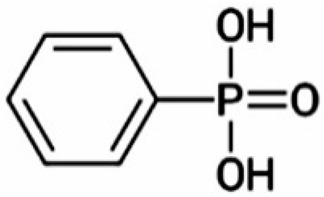	Molecular weight: 158.09 Melting point: 162–166 °C Density: 1.412 g/mL at 25 °C
3	Nitrilotrimethylphosphonic acid (ATMP)	C_3_H_12_NO_9_P_3_ 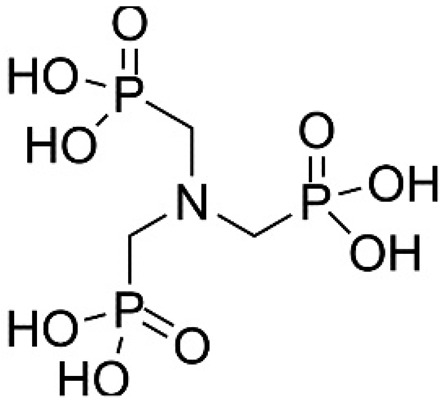	Molecular weight: 299.05 Melting point: 200 °C Density: 1.33 g/cm^3^ at 20 °C

**Table 2:** Elemental composition of HEDP+ Al_2_O_3_ microcapsules (activated).

Element	Weight, %	Atomic, %
C	28.48	39.27
O	39.92	41.33
Al	31.41	19.28
Si	0.19	0.11

**Table 3:** Calculated parameters of the system elements in the low-frequency region after exposure to 3% NaCl.

Exposure Time, days	R_1_, kOm·cm^2^	CPE_1_ Q0 μF·cm^2^	CPE_1_ n	R_2_, kOm·cm^2^	CPE_2_ Q0 μF·cm^2^	CPE_2_ n	W, kOm·cm^2^/s^0.5^
Primer Coating Comparison Sample							
1	0.3	0.017	1.00	14.3	2.551	0.56	13.8
2	0.9	0.016	1.00	14.2	1.414	0.64	12.9
15	1.0	0.022	1.00	15.6	1.372	0.56	8.8
21	10.7	2.134	0.49	18.6	226.400	1.00	9.4
42	1.3	4.013	0.46	7.5	884.370	1.00	6.2
91	1.4	0.075	1.00	4.6	181.956	1.00	7.1
Coating with 1% microcapsules of HEDP + Al_2_O_3_ (activated) composition							
1	8.10	0.341	0.57	56.55	0.332	0.80	15.16
2	8.68	0.073	0.66	118.22	0.340	0.70	27.39
15	6.19	0.010	0.87	130.57	0.262	0.72	32.61
21	16.28	0.055	0.65	171.53	0.217	0.70	18.60
42	15.93	0.131	0.60	146.79	0.262	0.68	13.56
91	7.08	0.007	1.00	363.73	0.380	0.49	17.65

## Data Availability

Data are contained within the article.
